# De novo tryptophanase-based indole production by metabolically engineered *Corynebacterium glutamicum*

**DOI:** 10.1007/s00253-023-12397-4

**Published:** 2023-02-14

**Authors:** Melanie Mindt, Lenny Ferrer, Dirk Bosch, Katarina Cankar, Volker F. Wendisch

**Affiliations:** 1 Wageningen Plant Research, Business Unit Bioscience, Wageningen University & Research, Wageningen, The Netherlands; 2 Axxence Aromatic GmbH, Emmerich am Rhein, Germany; 3 Genetics of Prokaryotes, Faculty of Biology & CeBiTec, Bielefeld University, Bielefeld, Germany; 4 Present Address: Translational Pharmacology, Faculty of Medicine OWL, Bielefeld University, Bielefeld, Germany

**Keywords:** *Corynebacterium glutamicum*, Indole, Tryptophanase, Microbial fermentation

## Abstract

**Abstract:**

Indole has an increasing interest in the flavor and fragrance industry. It is used in dairy products, tea drinks, and fine fragrances due to its distinct floral odor typical of jasmine blossoms. The current production of indole based on isolation from coal tar is non-sustainable and its isolation from plants is often unprofitable due to low yields. To offer an alternative to the conventional production, biosynthesis of indole has been studied recently. A glucose-based indole production was achieved by employing the *Corynebacterium glutamicum* tryptophan synthase α-subunit (TrpA) or indole-3-glycerol phosphate lyase (IGL) from wheat *Triticum aestivum* in a genetically-engineered *C. glutamicum* strain. In addition, a highly efficient bioconversion process using *C. glutamicum* heterologously expressing tryptophanase gene (*tnaA*) from *Providencia rettgeri* as a biocatalyst was developed. In this work, de novo indole production from glucose was enabled by expressing the *P. rettgeri tnaA* in a tryptophan-producing *C. glutamicum* strain. By metabolic engineering of a *C. glutamicum* shikimate accumulating base strain, tryptophan production of 2.14 ± 0.02 g L^-1^ was achieved. Introduction of the tryptophanase form *P. rettgeri* enabled indole production, but to low titers, which could be improved by sequestering indole into the water-immiscible solvent tributyrin during fermentation and a titer of 1.38 ± 0.04 g L^-1^ was achieved. The process was accelerated by decoupling growth from production increasing the volumetric productivity about 4-fold to 0.08 g L^-1^ h^-1^.

**Key points:**

• *Efficient de novo indole production via tryptophanases from glucose*

• *Increased indole titers by product sequestration and improved precursor supply*

• *Decoupling growth from production accelerated indole production*

## Introduction

The biotechnological workhorse *Corynebacterium glutamicum* is at the forefront of microbial-based bioeconomies. First isolated in 1956 by researchers of the Kyowa Hakko company in search of a natural glutamate producer (Kinoshita et al. 1957; Udaka 1960), this bacterium has been widely used in the multi-billion US$ market of glutamate and lysine (Lee & Wendisch 2017). With the advent of systems metabolic engineering and integrating genome editing with systems and synthetic biology, the product portfolio of *C. glutamicum* has been expanded to other amino acids (Hirasawa and Shimizu 2016; Lee and Wendisch 2017; Li et al. 2017; Ma et al. 2017; Wendisch 2020) and many other value-added compounds (Wolf et al. 2021). *C. glutamicum* possesses several intrinsic properties which makes it an advantageous choice as a production host. Among these merits is the relative ease of cultivation and the ability to grow fast to high cell densities (Hartbrich et al. 1996). In addition, it does not produce any endotoxins and its products are generally recognized as safe (GRAS). In the context of value-added aromatic compounds production, *C. glutamicum* has been shown to be more resistant to certain aromatics compared to other industrial microbial hosts such as *Escherichia coli*, *Pseudomonas putida*, and *Rhodobacter sphaeroides* (Kubota et al. 2016; Kitade et al. 2018). Hence, *C. glutamicum* is a sought-after production platform not only for amino acids but also for aromatic compounds. Production by interception of the bacterium’s native metabolic pathway of the aromatic amino acid tryptophan has also been shown (Katsumata and Ikeda 1993; Ikeda et al. 1994; Ikeda and Katsumata 1999).

While tryptophan itself has a great commercial value as a supplement in food and feed industry, its derivative indole is just as interesting. Indole is a bioactive aromatic compound which, at low concentration, exhibits a sweet and floral scent distinctive of a natural jasmine oil (Mookherjee et al. 1989; Edris et al. 2008). Essential oils of aromatic plants like jasmine are sought-after components in the flavor and fragrance industries due to their characteristic odor. Furthermore, indole serves as a precursor to the plant growth regulator indole-3-acetic acid, the dye indigo, and the pharmaceuticals melatonin and indole-3-carbinol (Barden 2010). Indole is mainly produced through three different methods; first, extraction from the non-renewable and unsustainable coal tar (Yamamoto et al. 1991; Kim 2019), second, isolation from flowering plants (Edris et al. 2008), which competes with arable land, and last, by chemical synthesis (Taber and Tirunahari 2011; Clarke et al. 2019) yielding indole classified as synthetic product, which is less desirable in the flavor and fragrance industries because it decreases its economic value (Braga et al. 2018). Hence, there is a demand for food-grade and environmentally conscious–sourced indole.

This can be achieved via microbial processes since several bacterial species, both Gram-positive and Gram-negative, are known to synthesize indole (Lee & Lee 2010). Production of flavors in microbes using fermentative processes leads to aroma compounds that are classified as natural by the European and US food legislation (Krings and Berger 1998; Paula Dionísio et al. 2012). Recently, bacterial genomes were explored by prospecting for *tnaA* genes (Mindt et al. 2022), encoding tryptophanases (TNA), which hydrolyze tryptophan in a β-elimination reaction and subsequently yield indole, pyruvate, and ammonia (Watanabe and Snell 1972). Mining of the bacterial sequence databases retrieved more than 400 candidates and subsequent in vivo screening in the host *C. glutamicum* strain showed that TNA from *Providencia rettgeri* supported indole production the best. Optimization of the process yielded an indole titer of 6 g L^-1^ in a bioconversion process from tryptophan. Kerbs et al. employed the TNA from *Escherichia coli* to produce indole and subsequently extended the synthetic pathway to indole alkaloids in *C. glutamicum* (Kerbs et al. 2022). A second indole producing process by *C. glutamicum* was based on the primary metabolism of bacteria and plants, which form indole during tryptophan biosynthesis (Ferrer et al. 2022a). Naturally, indole is an intermediate of the tryptophan biosynthesis, where it is channeled from the α-subunit to the β-subunit of the tryptophan synthase complex without its release. Plants overcome this limitation and produce indole by the activity of a different enzyme, indole-3-glycerolphosphate lyase, which forms indole from indole-3-glycerol-phoshate (IGP) as substrate. These enzymes presumably evolved from the tryptophan synthase α-subunit and act as stand-alone enzymes (Frey et al. 1997). Similarly, the tryptophan synthase α-subunit of *C. glutamicum* was shown to release indole when expressed in *C. glutamicum* in the absence of the β-subunit (Ferrer et al. 2022a) while the β-subunit alone condensed externally added indole with intracellularly synthesized serine to yield tryptophan (Ferrer et al. 2022b). Construction of an IGP-accumulating *C. glutamicum* strain by deletion of tryptophan synthase-encoding genes *trpBA* and plasmid-based expression of either indole-3-glycerolphosphate lyase from wheat or tryptophan synthase α-subunit from *C. glutamicum* enabled de novo production of 0.7 g L^-1^ indole from supplemented glucose and ammonium (Ferrer et al. 2022a).

Bioconversion (single reaction) or biotransformation (more than one reaction) processes are stereo- and regioselective chemical modifications of a precursor into a specific product catalyzed by biological systems such as cells or enzymes while de novo synthesis derives from simple building block molecules like sugars which are metabolized by the host organism to yield the desired product (Sales et al. 2018). In the bioconversion of tryptophan to indole, the cost of the substrate tryptophan plays an important role in determining the economic potential of the process. Therefore, in this study, indole was produced de novo by overexpressing *tnaA* from *P*. *rettgeri* in a *C. glutamicum* strain which was engineered to produce tryptophan.

In biotransformation, there is a stoichiometric requirement for the precursor tryptophan for indole production (e.g., 10 g/L tryptophan for 5.7 g/L indole (Mindt et al. 2022), while in the fermentation process developed here, the relatively cheaper carbon and nitrogen sources glucose and ammonium are used as substrates for de novo indole production. However, it has to be noted that the designed strain was auxotrophic for phenylalanine and tyrosine; thus, these amino acids had to be supplemented at 0.25 g L^-1^ each. As described here, the supplementation of only 0.25 g L^-1^ phenylalanine and 0.25 g L^-1^ tyrosine allowed for production of 1.4 g L^-1^ indole de novo, which to our knowledge is the best fermentation titer thus far.

## Materials and methods

### Bacterial strains and culture conditions

All bacterial strains and plasmids used in this study are listed in Tables 1 and 2. *E. coli* DH5α strain was used as a host for cloning (Hanahan 1983) and S17-1 for conjugation (Schäfer et al. 1994). Both strains were grown in Lysogeny Broth (LB; 10 g L^-1^ tryptone, 10 g L^-1^ sodium chloride, and 5 g L^-1^ yeast extract) at 37 °C. *C. glutamicum* C1*-derived strains were cultivated in Brain Heart Infusion (BHI) at 30 °C. When appropriate, culture medium was supplemented with 25 µg mL^-1^ kanamycin. Growth and production experiments with *C. glutamicum* were performed in CGXII minimal medium (Eggeling and Bott 2005) with 40 g L^-1^ glucose as carbon source, unless otherwise stated, and 1 mM IPTG (isopropyl-β-D-1-thiogalactopyranoside) for induction of gene expression from the pGold vector. For all strains derived from IND31, phenylalanine and tyrosine were supplemented to the minimal medium to a final concentration of 0.25 g L^-1^ each. For standard cultivations in minimal medium, overnight cultures of *C. glutamicum* in BHI were harvested and washed once with TN buffer (50 mM Tris-HCl, 50 mM NaCl, pH 6.3) before inoculation to an optical density at 600 nm (OD_600_) of 1 in 50 mL minimal medium. Growth in 500-mL baffled shake flasks was followed by measuring the OD_600_ using V-1200 spectrophotometer (VWR, Radnor, PA, USA). In situ product recovery experiments were performed exclusively in normal non-baffled flasks at 250 rpm. For growth-arrested cell reaction experiments, seed cultures in BHI and main cultures in CGXII of *C. glutamicum* IND330 and IND331 strains were cultivated as described previously. Main cultures were harvested 30 h after inoculation and transferred to fresh CGXII medium. Bacterial growth was prevented by omitting phenylalanine and tyrosine in the medium. Ten percent wet cell weight of IND330 and IND331 per culture volume were transferred to the fresh non-growth CGXII medium and incubated in non-baffled flasks at 250 rpm.

**Table 1:** Bacterial strains used in this study

Strain name	Description	Reference
ARO09	C1* Δ*vdh::*P_*ilvC*_*-aroG*^D146N^ Δ*ldhA* Δ*aroR::*P_*ilvC*_*-*_RBSopt_*aroF* Δ*qsuABCD::*P_*tuf*_*-qsuC* Δ*ppc::*P_*sod*_*-aroB* ΔP_*tkt*_*::*P_*tuf*_*-tkt* Δ*iolR*::P_*tuf*_*-aroE*	(Walter et al. 2020)
IND300	ARO09 carrying plasmid pGold-*trpE*^S40F^*D*_*EC*_	This study
IND301	ARO09 carrying plasmid pGold-*tnaA*_*Pre*_-*trpE*^S40F^*D*_*Ec*_	This study
IND31	ARO09 Δ*csm*	This study
IND310	IND31 carrying plasmid pGold-*trpE*^S40F^*D*_*EC*_	This study
IND311	IND31 carrying plasmid pGold-*tnaA*_*Pre*_-*trpE*^S40F^*D*_*Ec*_	This study
IND32	ARO09 Δ*csm* Δ*yggB*	This study
IND320	IND32 carrying plasmid pGold-*trpE*^S40F^*D*_*Ec*_	This study
IND321	IND32 carrying plasmid pGold-*tnaA*_*Pre*_-*trpE*^S40F^*D*_*Ec*_	This study
IND33	ARO09 Δ*csm* Δ*yggB* Δ*trpL*::P_*ilvC-*M1_*trpE*^S38R^	This study
IND330	IND33 carrying plasmid pGold-*trpE*^S40F^*D*_*Ec*_	This study
IND331	IND33 carrying plasmid pGold-*tnaA*_*Pre*_-*trpE*^S40F^*D*_*Ec*_	This study
IND332	IND33 carrying plasmid pGold-*tnaA*_*Pre*_-*trpE*^S40F^*D*_*Ec*_*-aroP*_*Cg*_	This study
IND3300	IND33 carrying plasmid pGold-empty	This study

**Table 2:** Plasmids used in this study

Plasmids	Description	Reference
pK19*mobsacB*	Km^R^, *E. coli*/*C. glutamicum* shuttle vector for construction of insertion and deletion mutants in *C. glutamicum* (pK18 *oriV*_*Ec*_* sacB lacZα*)	(Schäfer et al. 1994)
pK19-Δ*csm*	pK19*mobsacB* derivative for deletion of *csm* (cg0975)	(Li et al. 2009)
pK19 Δ*yggB*	pK19*mobsacB* derivative for deletion of *yggB* (cg1434)	(Pérez-García et al. 2019)
pK19-Δ*trpL::*P_*ilvC*-M1_*trpE*^S38R^_*Cg*_	pK19*mobsacB* derivative for replacement of *trpL* with *ilvC* promoter and simultaneous single-point S38R mutation in chromosomal native *C. glutamicum trpE*	(Veldmann et al. 2019)
pGold	Km^R^, P_*trc*_*lacI*^q^, pGA1, *oriV*_*Ec*_, *C. glutamicum*/*E. coli* expression shuttle vector with BsaI recognition site for Golden Gate assembly	(Walter et al. 2020)
pGold-*trpE*^S40F^*D*_*Ec*_	pGold expressing *trpE*^S40F^ and *trpD* from *E. coli* MG1655	(Ferrer et al. 2022a)
pGold-*tnaA*_*Pre*_-*trpE*^S40F^*D*_*Ec*_	pGold expressing *tnaA* from *Providencia rettgeri, trpE*^S40F^ and *trpD* from *E. coli* MG1655	This study
pGold-*tnaA*_*Pre*_-*trpE*^S40F^*D*_*Ec*_-*aroP*_*Cg*_	pGold expressing *tnaA* from *P. rettgeri, trpE*^S40F^, *trpD* from *E. coli* MG1655, and native *aroP* from *C. glutamicum* with an artificial RBS	This study

### Molecular genetic techniques and *strain construction*

Standard molecular methods including plasmid isolation, cloning, and transformation of plasmid DNA to *E. coli* by heat shock and to *C. glutamicum* by electroporation at 2.5 kV, 200 Ω, and 25 µF were performed as described elsewhere (Eggeling & Bott 2005; Hanahan 1983). The oligonucleotides used in this study are listed in Table 3. Golden Gate cloning strategy (Engler et al. 2008) with BsaI as type IIS restriction enzyme (New England Biolabs (NEB), Frankfurt, Germany) was employed to construct the plasmid pGold-*tnaA*_*Pre*_-*trpE*^S40F^*D*_*Ec*_. The plasmid pGold-*tnaA*_*Pre*_-*trpE*^S40F^*D*_*Ec*_-*aroP*_*Cg*_ was constructed by BamHI-digestion of pGold-*tnaA*_*Pre*_-*trpE*^S40F^*D*_*Ec*_ and inserting the amplified native *aroP* of *C. glutamicum* by Gibson assembly (Gibson et al. 2009). The gene *aroP* was amplified with ALLin^TM^ HiFi DNA Polymerase according to manufacturer (HighQu, Kraichtal, Germany) using *C. glutamicum* gDNA as template and aroP_fw and aroP_rv as primers. Gene deletions and insertions were performed using the suicide vector pK19*mobsacB*-derived plasmids in Table 2 by trans-conjugation with *E. coli* S17-1 as donor strain (Schäfer et al. 1994).

**Table 3:** Oligonucleotides used as primers in this study

Primer name	Sequence (5′-3′)	Description
aroP_fw	GGCACGAGGGTAAGTATGAG**GAAAGGAGGCCCTTCAGA**TGGCTAAATCTAATGAAGGGCTGGGAACC	Insertion of *aroP*_*Cg*_ in pGold-*tnaA*_*Pre*_-*trpE*^S40F^*D*_*Ec*_
aroP_rv	GAGGATCCCCGGGTACCGAGTCAGTTCAAGTCGGAAGGGGTGCG	
csm_fw	CGAAGCCTGCTCTGATAC	verification of *csm* deletion
csm_rv	GGCGTCGTTGATGATGTG	
trpL_fw	AGAATTCAGGATGAATTACTCGCTGGAATATTGGTG	verification of Δ*trpL*::P_*ilvC*‑M1_*trpE*^S38R^
trpL_rv	CTCGACAGCGGGGAGCGTTTC	
yggB_fw	GTCACTGGCATGGTGATGCCGC	verification of *yggB* deletion
yggB_rv	GCCAAAGGGCGCGAGCG	

Binding regions of Gibson primers are underlined and the RBS sequence is shown in bold.

### Analytical methods

The concentration of anthranilate, indole, and tryptophan was measured using reversed-phase high performance liquid chromatography (HPLC; 1200 series, Agilent Technologies Deutschland GmbH, Böblingen, Germany) equipped with Diode Array Detector (DAD, 1200 series, Agilent Technologies, Santa Clara, CA 95051, USA). Sample cell cultures were collected at different time points, followed by centrifugation at 14,000 rpm for 10 min. Supernatants were harvested and stored at -20 °C prior to analysis. Separation of aromatic compounds in the aqueous phase was performed with a pre-column [LiChrospher 100 RP18 EC-5µ (40×4 mM), CS Chromatographie Service, GmbH, Langerwehe, Germany] and a main column [LiChrospher 100 RP18 EC-5µ (125×4 mM), CS Chromatographie Service, GmbH] following the separation method as described by (Walter et al. 2020). Production titers were evaluated by using authentic standard curves as detected by DAD at 280 nm or 330 nm. Analysis of indole captured in tributyrin was carried out on a Waters HPLC system (e2695, Waters, Milford, Massachusetts, USA) using an RP18 column (Luna RP 18 3µ; 150×2 mm, Phenomenex) equipped with two pre-columns at a flow rate of 0.19 mL min^-1^ with 5 µL injection volume. The separation and detection method are described elsewhere (Ferrer et al. 2022a).

When indicated, Kovac’s assay was carried out for indole quantification according to the manufacturer. To prepare the standard linear curve, indole standard was dissolved in tributyrin. A total volume of 1,000 µL was used for the colorimetry at 571 nm. Assay solutions contained 980 µL of Kovac’s reagent (Merck, Darmstadt, Germany) and 20 µL of either indole standard or fermentation sample. Tributyrin was used as diluent when needed.

## Results

### Platform strain construction for de novo indole production

**Fig. 1:**
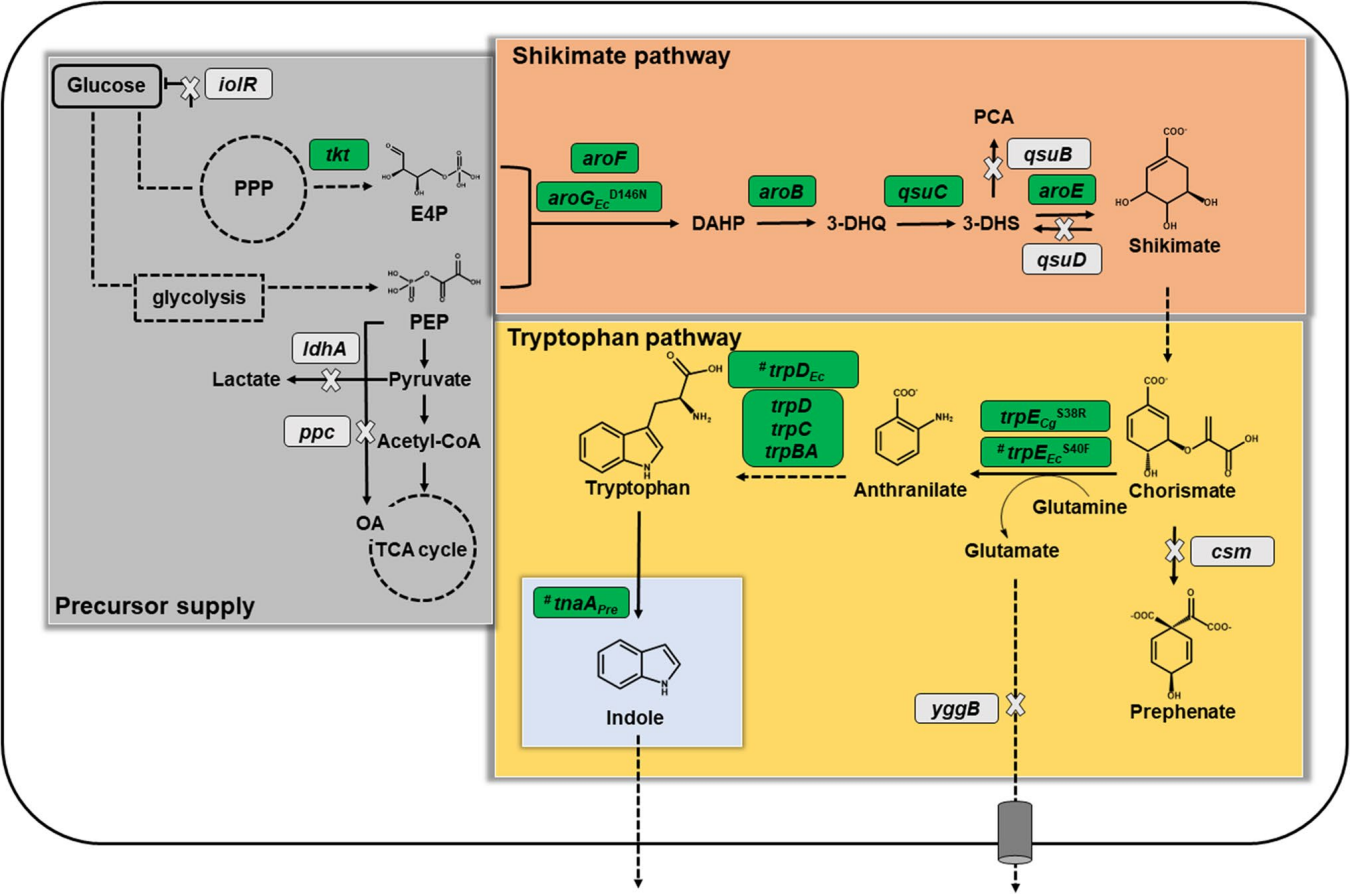
Schematic representation of metabolic engineering of *C. glutamicum* for indole production. Overexpressed genes are boxed in green while deleted genes are boxed in gray. Genes depicted in green indicate genome-based overexpression unless indicated by ^#^ which indicates vector-based expression. Strain engineering strategy was divided into three modules: (1) precursor supply, (2) shikimate pathway, (3) tryptophan pathway. *iolR*, IolT1 transcriptional regulator; PPP, pentose phosphate pathway; *tkt*, transketolase; E4P, erythrose-4-phosphate; PEP, phosphoenolpyruvate; *ldhA*, lactate dehydrogenase; *ppc*, PEP carboxylase; OA, oxaloacetate; TCA cycle, tricarboxylic acid cycle; DAHP, 3-deoxy-d- arabinoheptulosonate-7-phosphate; *aroF*, DAHP synthase; *aroG*_*Ec*_^D146N^, feedback-resistant DAHP synthase from *E. coli*; *aroB*, 3-dehydroquinate synthase; 3-DHQ, 3-dehydroquinate; *qsuC*, 3-dehydroquinate dehydratase; 3-DHS, 3-dehydroshikimic acid; PCA, protocatechuate; *qsuB*, 3-dehydroshikimate dehydratase; *qsuD*, shikimate dehydrogenase; *aroE*, shikimate dehydrogenase; *trpE*_*Cg*_^S38R^, feedback-resistant anthranilate synthase from *C. glutamicum*; *trpE*_*Ec*_^S40F^, feedback-resistant anthranilate synthase from *E. coli*; *csm*, chorismate mutase; *yggB*, MscS-type mechanosensitive channel; *trpD*, anthranilate phosphoribosyltransferase; *trpD*_*Ec*_, TrpD from *E. coli*, *trpC*, *N*-(5′-phosphoribosyl)anthranilate isomerase; *trpBA*, tryptophan synthase; *tnaA*_*Pre*_, tryptophanase from *Providencia rettgeri*.

To construct a functional de novo indole biosynthetic pathway in *C. glutamicum* based on TNA, ARO09, engineered for an increased shikimate pathway flux (Walter et al. 2020), was chosen as base strain and was further engineered to improve intracellular flux to the indole precursor tryptophan. To this end, the chorismate mutase-encoding *csm* gene was deleted in strain ARO09 to abolish the loss of chorismate towards phenylalanine and tyrosine, thus, increasing chorismate availability for the tryptophan pathway (Fig. 1). The *csm* deletion mutant IND31 and the parent strain ARO09 were transformed with either pGold-*trpE*^S40F^*D*_*Ec*_ (Ferrer et al. 2022a) or pGold-*tnaA*_*Pre*_-*trpE*^S40F^*D*_*Ec*_ to evaluate production of anthranilate, tryptophan, and indole. The native anthranilate synthase and anthranilate phosphoribosyltransferase of *C. glutamicum* encoded by *trpE* and *trpD,* respectively, are feedback inhibited by tryptophan and its derivatives with the latter having a K_*i*_ of 0.83 mM (0.17 g L^-1^) tryptophan (O’Gara and Dunican 1995). Therefore, feedback-resistant variant *trpE*^S40F^ (Caligiuri and Bauerle 1991) and *trpD* from *E. coli* were overexpressed to allow accumulation of tryptophan by the host strain. Previously, it was demonstrated that overexpression of *trpE*^S40F^ from *E. coli* results to anthranilate accumulation in *C. glutamicum* C1* (Walter et al. 2020).

**Fig. 2:**
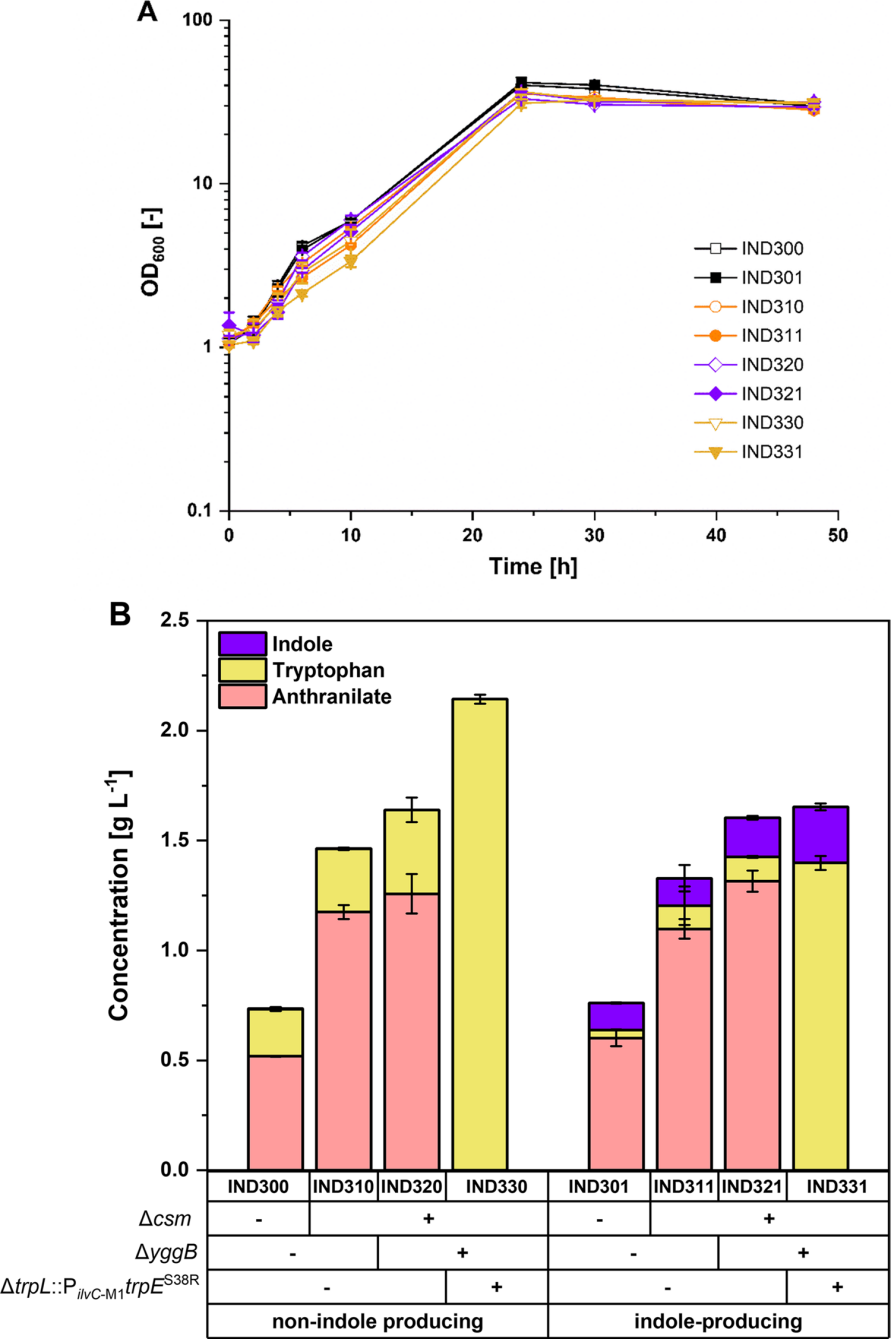
Growth (**A**) and production of indole and precursors (**B**) in consecutively constructed IND strains. Cultivation was done in shake flasks with CGXII minimal medium and 40 g L^-1^ glucose, supplemented with 0.25 g L^-1^ tyrosine and phenylalanine. Genetic modifications performed in ARO09 strain to yield corresponding IND strains are indicated by (+) or (−) to indicate presence or absence of the modification, respectively. Non-indole producing strains carry the plasmid pGold-*trpE*^S40F^*D*_*EC*_ while indole-producing strains contain the plasmid pGold-*tnaA*_*Pre*_-*trpE*^S40F^*D*_*Ec*_. Concentrations from supernatant samples were determined after 48 h and are given as means and standard deviations of three independent cultures.

The *csm* deletion mutant harboring pGold-*trpE*^S40F^*D*_*Ec*_ (i.e., IND310; Table 1) showed similar growth rates when supplemented with phenylalanine and tyrosine than IND300 (i.e., IND300; Table 1, Fig. 2) and produced 1.18 ± 0.03 g L^-1^ anthranilate, 2.26-fold more than that of the base strain without *csm* deletion carrying the same plasmid after 48 h of cultivation in CGXII medium and 40 g L^-1^ glucose. Both IND300 and IND310 strains also accumulated tryptophan to titers of 0.21 ± 0.01 g L^-1^ and 0.29 ± 0.01 g L^-1^, respectively. To enable hydrolytic β-elimination of tryptophan to indole, we chose the previously characterized TNA from *P. rettgeri* which allowed efficient conversion of tryptophan to indole in a bioconversion process (Mindt et al. 2022). Hence, the plasmid pGold-*tnaA*_*Pre*_-*trpE*^S40F^*D*_*Ec*_ was constructed and used to transform strains ARO09 and IND31 yielding IND301 and IND311, respectively. Overexpression of *tnaA*_*Pre*_ allowed indole production, albeit to similar concentrations of around 0.12 g L^-1^ for both IND301 and IND311 (Fig. 2B). Thus, de novo indole pathway based on TNA was established in *C. glutamicum* and subsequent experiments were performed to either further increase precursor supply or resolve bottlenecks to achieve full indole conversion.

### Anthranilate production using gluconate or sucrose as the carbon source

**Fig. 3:**
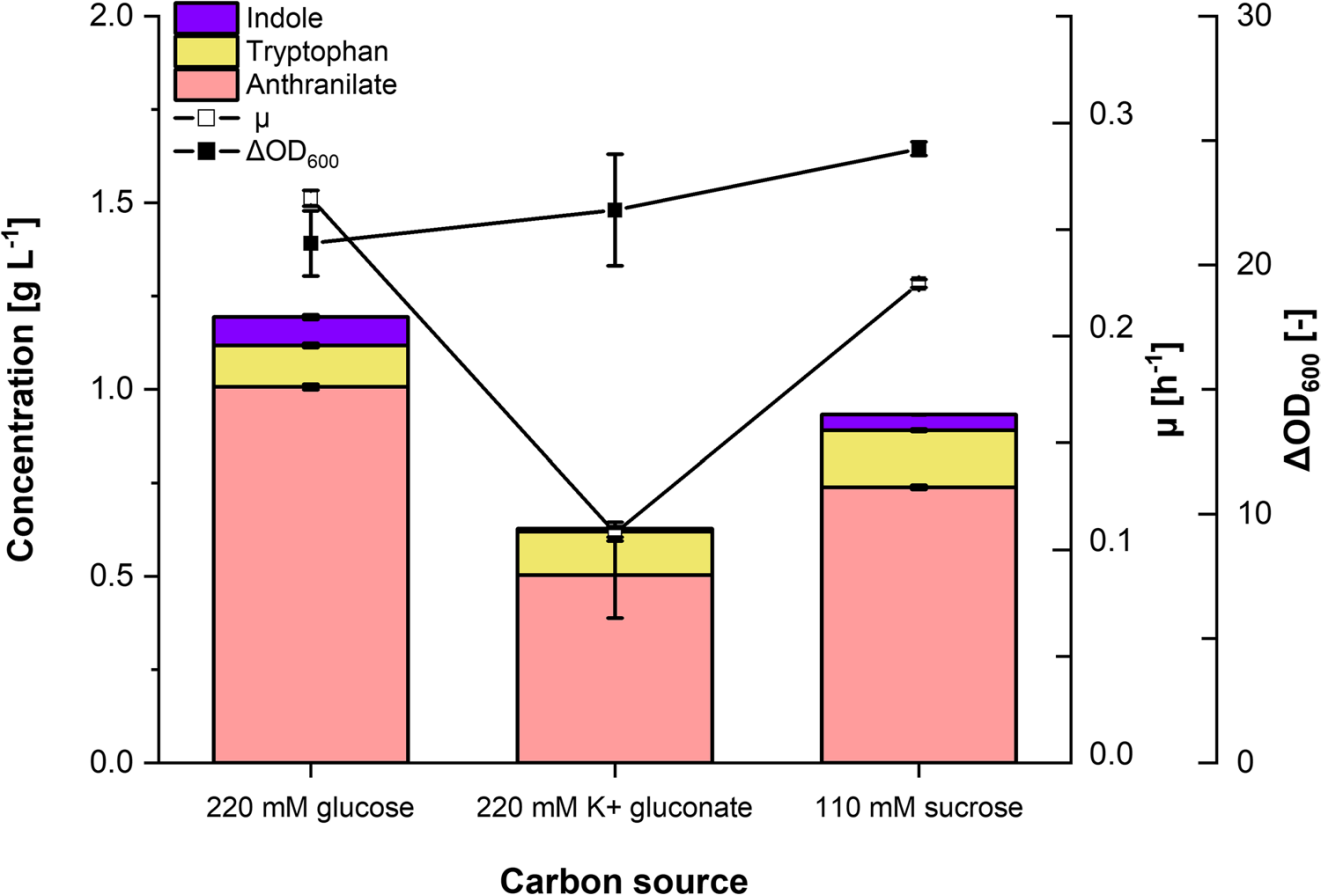
Production of anthranilate, tryptophan, and indole by *C. glutamicum* strain IND311 with different carbon sources. After growing IND311 for 48 h in a BioLector microcultivation system with either 220 mM glucose, 220 mM K^+^ gluconate, or 110 mM sucrose as carbon source, the concentrations of anthranilate, tryptophan, and indole were determined in culture supernatants. Means and standard deviations of three independent cultures are shown.

The majority of biotechnological processes rely on glucose as carbon source because of its low cost. In *C. glutamicum*, it is mainly imported with concomitant phosphorylation through PTS^Glc^ (Moon et al. 2007). Assimilation of glucose by PTS^Glc^ requires the glycolytic intermediate phosphoenolpyruvate (PEP) as phosphoryl group donor, resulting in reduced intracellular PEP level and thus competes with PEP availability as a precursor to PEP-derived products like l-lysine, succinic acid, and, of interest in this study, aromatic compounds (Ruan et al. 2020). Therefore, varying the carbon source from a PTS sugar, i.e., glucose, to a non-PTS carbon source, i.e., gluconate, may increase the provision for the precursor PEP and may subsequently lead to increased products formation. In order to investigate the effect of using a non-PTS sugar as a carbon source, IND311 strain was cultivated with either 220 mM glucose or 220 mM K^+^ gluconate in a BioLector microcultivation system. After 48 h of cultivation, accumulated anthranilate in the supernatant using gluconate as carbon source to only half of the concentration obtained with glucose, i.e., 0.50 ± 0.12 and 1.01 ± 0.01 g L^-1^, respectively, while tryptophan was produced to around 0.11 g L^-1^ for both carbon sources (Fig. 3). Furthermore, indole produced from gluconate was less at 0.01 ± 0.01 g L^-1^ compared to 0.08 ± 0.01 g L^-1^ obtained from glucose. Additionally, although IND311 grew to a comparable biomass in both gluconate and glucose, this strain grew slower and had lower maximal growth rate. Accordingly, glucose was superior in both production and growth performance than gluconate.

Anthranilate and other amine derivatives such as *para*-aminobenzoate or *meta*-aminobenzoate were observed to undergo a non-enzymatic *N*-glycation reaction with the aldehyde group of glucose (Kubota et al. 2016). As a consequence, *para*-aminobenzoate titer was reduced when produced via fermentation with glucose as carbon source, but not with sucrose (a disaccharide without an aldehyde group). In order to determine if using glucose to grow the host strain limits anthranilate titers due to *N*-glycation, 220 mM glucose was replaced with 110 mM sucrose in the CGXII minimal medium. IND311 produced 0.74 ± 0.01 g L^-1^ anthranilate after 48 h culture in BioLector, lower than the 1.01 ± 0.01 g L^-1^ produced from glucose. This result suggests that *N*-glycation is not a bottleneck to achieving higher anthranilate titers in the current process.

### Further strain optimization and releasing bottleneck of converting anthranilate to tryptophan

Deletion of the glutamate exporter *yggB* was shown to be helpful in increasing the anthranilate titer (Ferrer et al. 2022a), which is probably due to reduction of glutamate excretion (Nakamura et al. 2007) since glutamate is released in the TrpE reaction. Glutamate is required to regenerate glutamine by the glutamine synthetase I encoded by *glnA* since the amide nitrogen is the source of the amino group of anthranilate in the TrpE enzymatic reaction (Bakers and Crawford 1966). Hence, native *yggB* was deleted from the IND31 genome resulting in strain IND32. Transformation of IND32 with pGold-*tnaA*_*Pre*_-*trpE*^S40F^*D*_*Ec*_ yielded strain IND321, which produced 1.32 ± 0.05 g L^-1^ anthranilate after 48 h of cultivation, more than that produced by the parental strain IND311 (Fig. 2B). Noteworthy, however, is that the increase in anthranilate titer did not directly correlate with the increase in either tryptophan or indole.

Strain IND321 accumulated the precursor anthranilate in a gram per liter scale. However, tryptophan production remained minimal despite plasmid-based overexpression of *trpD* from *E. coli*. Therefore, to further enforce the carbon flow from anthranilate to tryptophan, the gene for the leader peptide *trpL* (Sano and Matsui 1987; Seliverstov et al. 2005) was deleted in IND32 to alleviate the attenuation control of endogenous *trp* operon. Single-base exchange was performed simultaneously to introduce the S38R mutation to the native TrpE as it is known to cause the desensitation of native TrpE from tryptophan feedback inhibition (Matsui et al. 1987). The resulting strain was named IND33. Either the empty vector pGold or pGold-*trpE*^S40F^*D*_*Ec*_ were used to transformed IND33 to yield strains IND3300 and IND330, respectively. Shake flask cultivation of these two strains demonstrated two interesting results. First, anthranilate production by IND3300 was increased to 2.04 ± 0.05 g L^-1^ even without overexpression of *trpE*^S40F^*D* from *E. coli* as consequence of the amino acid exchange S38R of native TrpE. However, the introduced point mutation and the release of attenuation control of the *trp* genes were not sufficient to convert anthranilate to tryptophan (i.e., IND3300 harboring only the empty vector pGold). In strain IND330 where *trpE*^S40F^*D* from *E. coli* was expressed in addition, no anthranilate was detected and tryptophan was produced at a titer of 2.14 ± 0.02 g L^-1^ (Fig. 2B). After successfully easing the bottleneck from anthranilate to tryptophan, the next hurdle to be solved was converting the precursor tryptophan to the product indole.

### Efficient fermentative production of indole from tryptophan

To evaluate the potential of the engineered strain IND33 to convert tryptophan to indole, pGold-*tnaA*_*Pre*_-*trpE*^S40F^*D*_*Ec*_ was used to transform IND33, named IND331. In a glucose and ammonium based medium, IND331 produced 0.26 ± 0.02 g L^-1^ (2.2 ± 0.2 mM) indole despite copious availability of its precursor as 1.40 ± 0.03 g L^-1^ (6.9 ± 0.2 mM) of tryptophan was secreted (Fig. 2B). This result contrasts with the previous observation in *E. coli* that 5 mM tryptophan added to the medium were almost completely converted to indole (4.7 mM) when endogenous *tnaA* and tryptophan import gene *tnaB* were overexpressed (Li & Young 2013). Plasmid-based overexpression of either transporter genes *tnaB* from *E. coli* or endogenous aromatic amino acid importer *aroP* (Wehrmann et al. 1995) both resulted in decreased tryptophan concentrations when added in the medium, but the latter showed better substrate conversion and indole production (Mindt et al. 2022). Since the tryptophan exporter gene(s) remain unknown in *C. glutamicum*, blocking the secretion of tryptophan is not possible. As alternative, improving re-import of tryptophan into the cell was tested. Hence, the plasmid pGold-*tnaA*_*Pre*_-*trpE*^S40F^*D*_*Ec*_-*aroP*_*Cg*_ was constructed to facilitate re-uptake of tryptophan into the cell for further conversion to indole. Strain IND33 harboring this plasmid was named IND332.

**Fig. 4:**
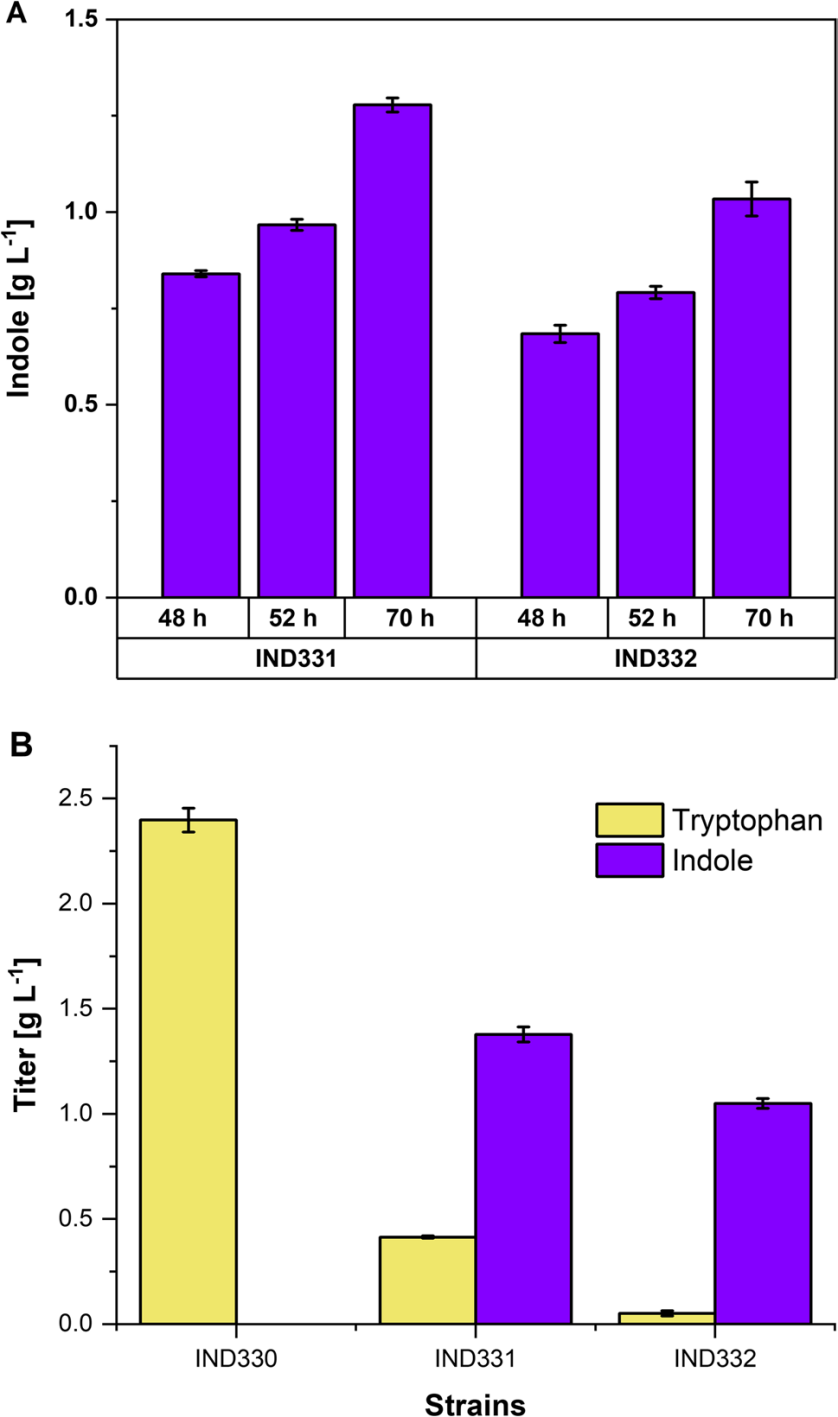
In situ extractive shake flask cultivation in non-baffled flasks for indole production to address indole toxicity. Production of aromatic compounds by IND330, IND331, and IND332 strains was determined by Kovac’s assay for indole (**A**) and by HPLC for tryptophan and indole (**B**). Samples for tryptophan and indole determination by HPLC analysis were obtained from the aqueous layer and tributyrin layer, respectively, after 70 h of cultivation. Values and error bars represent mean and data range of duplicate cultivations.

It is known that 3 to 5 mM (0.35 to 0.58 g L^-1^) indole is a potentially growth inhibitory concentration in bacteria as it presumably reduces the electrochemical potential across the cytoplasmic membrane (Chimerel et al. 2012) and indole toxicity was successfully overcome in *C. glutamicum* by in situ two-phase extractive fermentation using dibutyl sebacate or tributyrin as extractants, which enabled complete tryptophan conversion up to indole product titer of 5.7 g L^-1^ in a bioconversion process (Mindt et al. 2022) and 0.7 g L^-1^ indole in a de novo process from glucose by IGL enzyme (Ferrer et al. 2022a). Hence, this concept was applied to TNA-based de novo production of indole and the tryptophan producer IND330 and indole producers IND331 and IND332 were grown in CGXII medium with 20% (vol/vol) tributyrin as overlay. Under these conditions, tryptophan remains in the aqueous phase and does not partition to the tributyrin phase which completely sequestered indole (Ferrer et al. 2022a; Mindt et al. 2022). Accordingly, tryptophan was quantified in the aqueous phase and indole in the tributyrin layer. Kovac’s assay showed that both IND331 and IND332 produced highest indole titers at 70 h (Fig. 4A). For quantification of tryptophan and indole, both aqueous and tributyrin samples collected at 70 h for all the three strains tested were subjected to HPLC analysis. Tryptophan was detected in the aqueous layer for both IND330 and IND331 at concentrations 2.40 ± 0.06 g L^-1^ and 0.41 ± 0.01 g L^-1^, respectively, but not in IND332 that overexpressed the importer gene *aroP*. Interestingly, IND331 produced 1.38 ± 0.04 g L^-1^ indole with a volumetric productivity of 0.02 g L^-1^ h^-1^, while IND332 revealed an indole titer of 1.05 ± 0.02 g L^-1^. Thus, *aroP* overexpression was not helpful in increasing the overall indole production and the best indole-producing strain, IND331, showed the highest de novo indole titer thus far, and was used in the next production experiments.

### Decoupling biomass formation with indole production

Since model simulations showed that indole production is vastly limited at maximum growth conditions (Mindt et al. 2022), two strategies were explored to decouple the biomass formation stage from the indole production phase. The first strategy was to reroute the carbon and other cellular resources towards product synthesis by titrating phenylalanine and tyrosine. Since strain IND331 is auxotrophic for these two aromatic amino acids, it was speculated that reducing their concentration in the medium will lead to less biomass formed and that the carbon from glucose will be instead more efficiently channeled towards product synthesis. Consequently, strain IND331 was grown in a deep-well plate using CGXII medium with 40 g L^-1^ glucose and supplemented with different concentrations of phenylalanine and tyrosine that varied from 0.025 g L^-1^ to 0.35 g L^-1^, each.

**Fig. 5:**
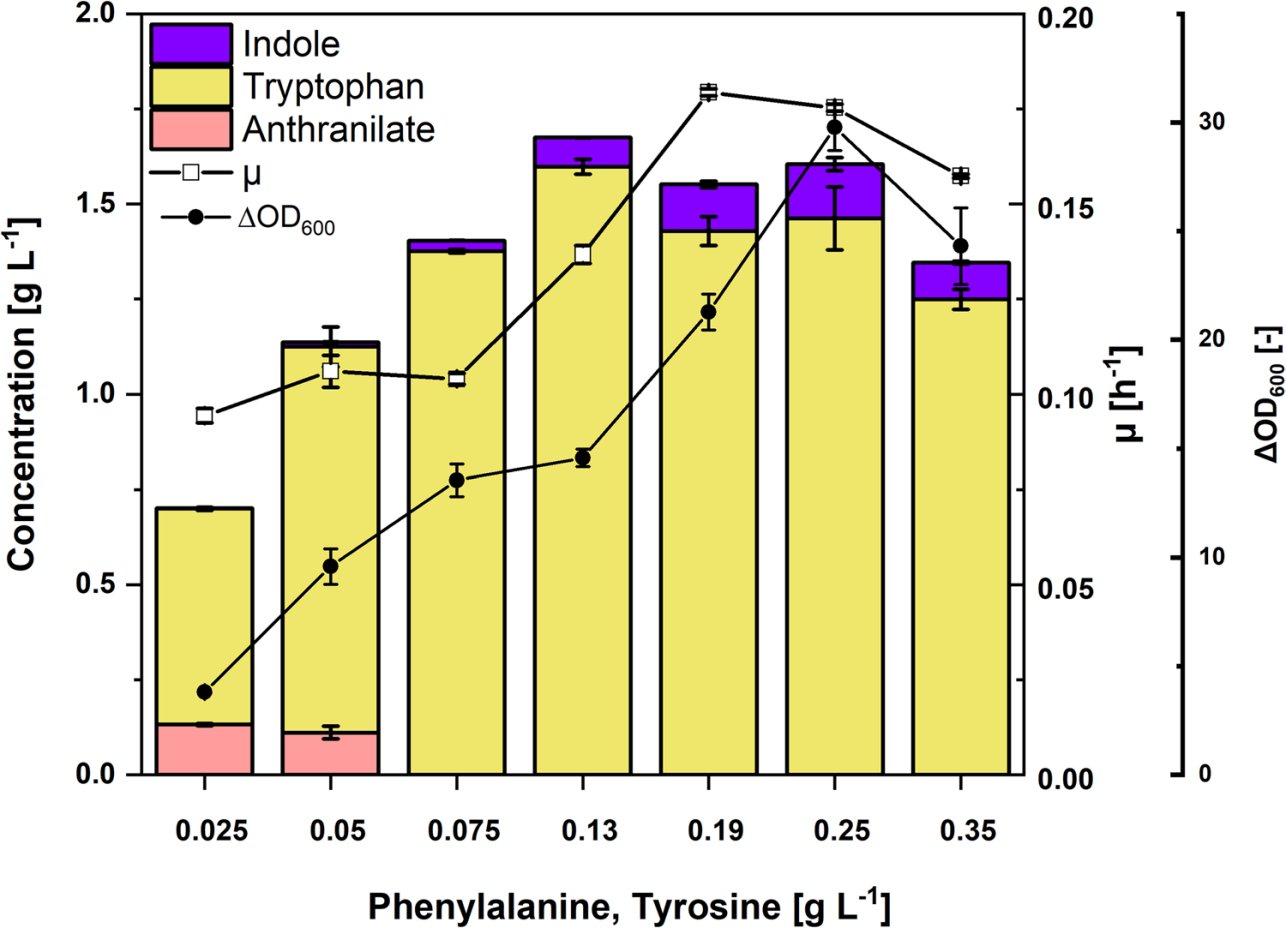
Production of anthranilate, tryptophan, and indole by *C. glutamicum* strain IND331 with varying phenylalanine and tyrosine concentrations as supplements. Titration of phenylalanine and tyrosine was performed by growing IND331 in a BioLector microcultivation system. Titers were determined after 48 h and are shown as means and standard deviation of three independent cultures. The biomass formed is given as ΔOD_600_, the growth rate in h^-1^.

The highest biomass formation of IND331 was observed upon addition of phenylalanine and tyrosine at concentrations of 0.25 g L^-1^ each. Reducing the supplementation of amino acids required for biomass formation led to a decrease in biomass formation, as expected. Interestingly, production of both tryptophan and indole at 0.25-, 0.19-, and 0.13-g L^-1^ phenylalanine and tyrosine supplementation did not vary significantly despite the lower biomass formed (Fig. 5). The carbon from glucose, however, was not successfully redirected towards product synthesis by this approach.

**Fig. 6:**
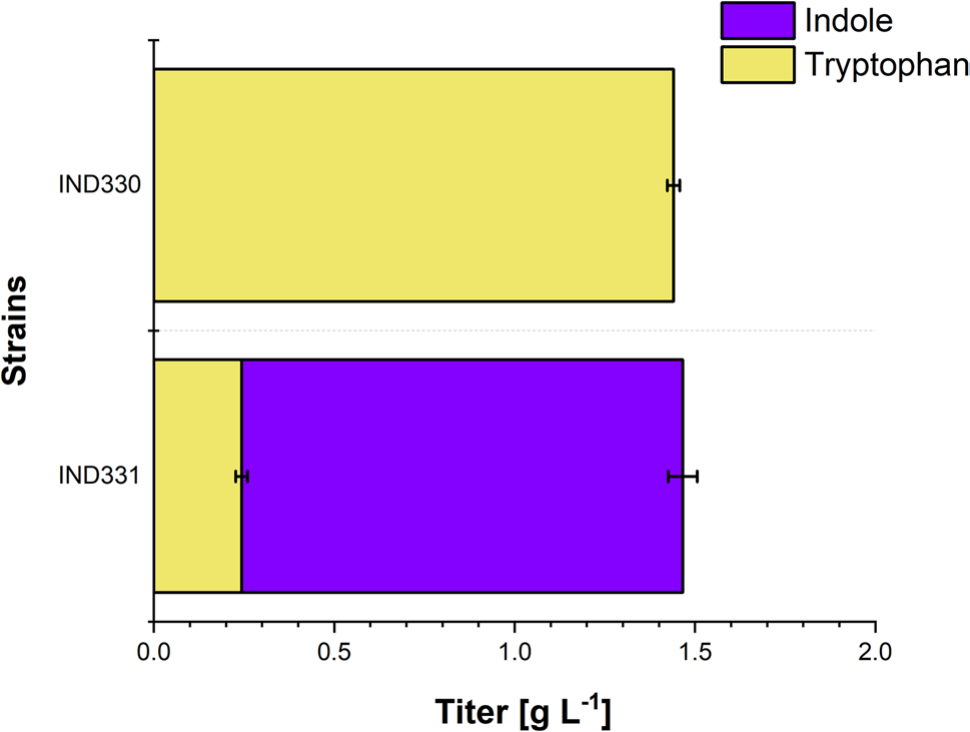
Production of tryptophan and indole by *C. glutamicum* strains IND330 and IND331 in a growth-arrested cell reaction with in situ product recovery. Tryptophan was quantified in the aqueous medium phase while indole was analyzed in the tributyrin layer. IND330 and IND331 were transferred to non-growth CGXII medium lacking phenylalanine and tyrosine to a final concentration of 10% wet cell weight per culture volume. Data are averages and standard deviations from three independent cultures.

As a second strategy indole production by aerobic growth-arrested cells was performed to decouple growth and production. Accordingly, pre-grown IND330 and IND331 cells were transferred to reach 10 % (w/vol) cell wet weight in minimal CGXII medium without phenylalanine and tyrosine. To this non-growth medium, 20% (vol/vol) tributyrin was added for indole sequestration. By combining growth-arrested production and in situ product recovery, IND331 produced 1.22 ± 0.04 g L^-1^ indole from 40 g L^-1^ glucose after 15 h (volumetric productivity of 0.08 g L^-1^ h^-1^ excluding seed culture; 0.03 g L^-1^ h^-1^ including seed culture). In addition, accumulation of 0.24 ± 0.02 g L^-1^ tryptophan was measured in the aqueous phase (Fig. 6). This result suggests that this combined strategy can reach similar indole titer in just 15 h compared to 70 h with in situ product recovery without growth arrest.

## Discussion

De novo indole production was achieved by applying the TNA enzyme from *P. rettgeri* in a metabolically engineered *C. glutamicum* tryptophan producer. The titer of about 1.4 g L^-1^ achieved in this study is higher than that obtained by de novo indole production based on IGL from wheat *T. aestivum* or TrpA from *C. glutamicum* which yielded 0.7 g L^-1^ (Ferrer et al. 2022a).

Compared to the platform strain ARO09 (Walter et al. 2020), here, provision of anthranilate was increased by the deletion of the *csm* gene resulting in higher anthranilate accumulation (compare strain IND310 to IND300, Fig. 2B). This strategy rendered the strain auxotrophic for phenylalanine and tyrosine. High cell density cultivations of this newly constructed strain and its ancestors will require an adapted supplementation of phenylalanine and tyrosine. To meet conditions in which glucose is mainly channeled to the desired product instead of biomass, precise balancing of their supplementation is crucial (Bartek et al. 2008). However, in contrast to this expected outcome, testing different supplementation concentrations in the BioLector using the best indole-producing strain IND331 reduced biomass formation, but did not increase indole production. Possibly, carbon from glucose was channeled to other metabolites. Thus, a detailed medium optimization needs to be performed including e.g., independent variation of the supplemented phenylalanine and tyrosine concentrations. Alternatively, instead of deletion of *csm*, its transcription and/or translation efficiency may be lowered by altering promoter and ribosome binding site sequences of *csm*.

In the conventional single-phase fermentation process where indole production is coupled to the growth of strain IND331, tryptophan accumulated up to 1.40 ± 0.03 g L^-1^ (6.9 ± 0.2 mM) after 48 h; nevertheless, indole titer remained minimal at 0.26 ± 0.02 g L^-1^ (2.2 ± 0.2 mM) (Fig. 2). Hence, the conversion of tryptophan to indole was 32 mol-% which was significantly lower than the 61 mol-% conversion observed in the bioconversion process wherein 0.35 g L^-1^ indole was produced from 1 g L^-1^ tryptophan within 24 h using strain C1* (pGold‑*PretnaA*-*CgaroP*) (Mindt et al. 2022). Notably, with in situ product recovery, the bioconversion process enabled complete molar conversion of 10 g L^-1^ tryptophan to indole within 24 h (Mindt et al. 2022). The rate of indole formation was much slower for the de novo process described here as strain IND331 produced 1.38 ± 0.04 g L^-1^ indole within 70 h (Fig. 4A). It was speculated that tryptophan secretion is faster compared to its conversion to indole, and as consequence not available as substrate for TNAs. Elimination of tryptophan secretion is not possible because the genes coding for tryptophan export have not yet been identified. Alternatively, upregulation of tryptophan import by expression of *aroP* was tested but did not accelerate nor improve the production. Tryptophan and indole secretion even dropped upon *aroP* expression. Due to deregulation of the endogenous *trp*-operon by deletion of *trpL* and introduction of feedback-resistant versions of the feedback-regulated DAHP synthase and shikimate dehydrogenase, downregulation of tryptophan biosynthesis as a consequence of *aroP* overexpression can be excluded. The lower production titer in IND332 could be explained by either diminished growth of the strain due to plasmid-based expression of a membrane protein encoding gene (Wagner et al. 2007), or by re-routing of tryptophan towards biomass formation instead of production. Noteworthy, however, is that due to addition of the second immiscible tributyrin layer, the growth of both IND331 and IND332 could not be accurately determined by conventional measurement of optical density at 600 nm. The differences in bioconversion and de novo production may also indicate that biomass formation and/or de novo biosynthesis of tryptophan and its subsequent conversion to indole were off-balanced. It has to be noted that at least some TNAs carry additional β-elimination activities, e.g., as cysteine desulfhydrases and deletion of *tnaA* in *E. coli* improved production of cysteine (Awano et al. 2003). Thus, growing cells producing TNA may be limited by cysteine availability for growth. Therefore, growth-decoupled production was tested. Notably, previous growth-arrested production strategies employing packed high-cell density cultures proved to be effective in improving volumetric productivity of aromatic compounds (Kogure et al. 2016; Kitade et al. 2018).

Indeed, decoupling biomass formation and indole production through growth-arrested cell reaction successfully improved the volumetric productivity as the process was considerably shortened from 70 to 15 h (excluding preculture of 30 h). The indole yield on glucose, however, remained comparable even though it was expected that such growth-arrested process will facilitate high yields due to channeling of energy formed from glycolysis to product formation instead of biomass generation (Jojima et al. 2013). Interestingly, shikimate, an indole precursor, is produced with high productivity and high yield under aerobic growth-arrested reaction, but not under anaerobic condition in which glucose consumption decreased and formation of organic acids as side products increased (Kogure et al. 2016). It was suggested that under aerobic conditions, NADH generated in glycolysis can be re-oxidized by oxygen and respiration, but under oxygen deprivation conditions, NADH cannot be re-oxidized by the shikimate biosynthetic pathway causing cofactor imbalance (Jojima et al. 2010; Hasegawa et al. 2012). Thus, it may be possible that the oxygen supply of the indole process described here was insufficient during the growth-arrested process, especially since non-baffled shaking flasks were employed. Non-baffled shaking flasks support less aeration, but had to be used to provide the uniform regular swirling liquid flow when a second non-immiscible layer is added to the medium for in situ product removal. Therefore, it may prove beneficial to perform a bioreactor cultivation as this setup provides good mixing, a high interphase area and the option to monitor and control the oxygen supply.

The proof-of-concepts for de novo indole processes using TNA, as described here, and IGLs (Ferrer et al. 2022a) need to be improved further to sustain high product titers, yields, and productivities. This will be relevant in the future as indole is not only used as an odorant and a flavor enhancer, but also serves as the precursor for the biosynthesis of other value-added chemicals such as the dye indigo, the plant growth regulator indole-3-acetic acid, and the pharmaceuticals melatonin and indole-3-carbinol. Especially when targeting high volume markets, de novo production of indole from cheap and sustainable carbon sources becomes important. In this study, glucose, the preferred carbon source of *C. glutamicum* (Wendisch et al. 2000), was used as the substrate. While glucose is relatively a cheaper substrate compared to tryptophan in the bioconversion process (Mindt et al. 2022), it is a first-generation feedstock which may potentially compete with food and animal feed. Therefore, implementing the flexible feedstock concept for indole production by *C. glutamicum* using non-edible lignocellulosic biomass may prove beneficial in increasing the industrial competence of the process. *C. glutamicum* has been extensively engineered to broaden its substrate spectrum to lignocellulosic pentoses arabinose and xylose for the production of amino acids (Schneider et al. 2011; Meiswinkel et al. 2013), less processed lignocellulosic residuals such as wheat sidestream (Burgardt et al. 2021), and of specific relevance to indole and other nitrogen-containing target products, nitrogenous sidestream from the fishery industry (Matano et al. 2014; Matano et al. 2016).

## Data Availability

All data generated or analyzed during this study are included in this published article.
